# *Cj0440c* Affects Flagella Formation and *In Vivo* Colonization of Erythromycin-Susceptible and -Resistant *Campylobacter jejuni*

**DOI:** 10.3389/fmicb.2017.00729

**Published:** 2017-04-25

**Authors:** Haihong Hao, Xia Fang, Jing Han, Steven L. Foley, Yulian Wang, Guyue Cheng, Xu Wang, Lingli Huang, Menghong Dai, Zhenli Liu, Zonghui Yuan

**Affiliations:** ^1^National Reference Laboratory of Veterinary Drug Residues and MOA Key Laboratory for Detection of Veterinary Drug Residues, Huazhong Agricultural UniversityWuhan, China; ^2^MOA Laboratory for Risk Assessment of Quality and Safety of Livestock and Poultry Products, Huazhong Agricultural UniversityWuhan, China; ^3^Division of Microbiology, National Center for Toxicological Research, US Food and Drug Administration, JeffersonAR, USA

**Keywords:** *Campylobacter jejuni*, Cj0440c, flagella, colonization, erythromycin resistance

## Abstract

*Campylobacter jejuni* is one of the most common foodborne pathogen worldwide. A putative transcriptional regulator, *Cj0440c*, was up-regulated in the erythromycin-resistant *C. jejuni*, however, the precise role of *Cj0440c* is yet to be determined. The aim of this study was to determine the biological functions of *Cj0440c*. The *Cj0440c* isogenic mutants were constructed from erythromycin-susceptible *C. jejuni* NCTC 11168 (S) and -resistant *C. jejuni* 68-ER (R), designating as SM and RM, respectively. The isogenic Cj0440c mutants (SM and RM) and parental strains (S and R) were subjected to microarray and qRT-PCR analysis to examine the transcriptional profile changes contributed by *Cj0440c*. The antimicrobial susceptibility, flagellar morphology, *in vitro* growth and *in vivo* colonization in chickens were carried out to analyze the biological function of *Cj0440c*. The results showed that 17 genes were down-regulated in SM compared to S, while 9 genes were down-regulated in RM compared to R. The genes with transcriptional change were mainly involved in flagella biosynthesis and assembly. Using transmission electron microscopy, we found that the filaments were impaired in SM and lost in RM. The chicken colonization experiments showed that *Cj0440c* mutants (SM and RM) had reduced colonization ability in chickens when compared with corresponding parental strains (S and R). In conclusion, *Cj0440c* regulates flagella biosynthesis and assembly, and consequently affect the *in vivo* colonization of erythromycin-susceptible and -resistant *C. jejuni*.

## Introduction

*Campylobacter jejuni* has been recognized as one of the most important pathogens, which can cause infectious diarrhea and severe forms of disease such as Guillain-Barre Syndrome or Miller Fischer Syndrome (Samuel et al., 2004; Hughes and Cornblath, 2005; Riddle et al., 2006). The CDC estimated that in 2009 the number of *Campylobacter* infection was 13.02 per 100,000 people (Silva et al., 2011). The cost of human Campylobacteriosis in the United States is estimated at $1.3 to 6.8 billion dollars annually (Scharff, 2012; Epps et al., 2013). Macrolides (e.g., erythromycin) are the most important drugs of choice for clinical treatment of *Campylobacter* infections (Gibreel et al., 2005). Unfortunately, macrolides-resistant *Campylobacter* have emerged and impose a global public health concerns (Gibreel and Taylor, 2006; ECDC et al., 2009). In earlier study we demonstrated that the transcription level of *Cj0440c* was increased in high-level erythromycin-resistant *C. jejuni* (Hao et al., 2013).

Bioinformatic analyses suggested that *Cj0440c* is a putative transcriptional regulator and encodes a TENA/THI-4 family protein, however, the molecular function of this family is yet to be determined. The gene *Cj0440c* is located downstream of the *Cj0437–Cj0439* operon *(mfr*, methylmenaquinol:fumarate reductase) which plays an important role in the susceptibility to hydrogen peroxide (H_2_O_2_) (Parkhill et al., 2000; Weingarten et al., 2009; Kassem et al., 2012) and upstream of *Cj0441* (*acpP*, acyl carrier protein) which is a universal and highly conserved acyl donor for synthesis of fatty acid, endotoxin and acylated homoserine lactones for the quorum sensing in *C. jejuni* (Byers and Gong, 2007). Both the downstream and upstream genes of *Cj0440c* were essential for the growth, survival, colonization and pathogenesis in *Campylobacter.* Although *Cj0440c* is located on the opposite DNA coding strand, it may divergently transcribed with its up-and-downstream genes, and likely to act as a transcriptional regulator and play an important role in gene regulation and the biological function in *C. jejuni*. The biological functions of *Cj0440c* in *C. jejuni* are largely unknown.

In the present study, *Cj0440c*-inactivated mutation was constructed in both erythromycin-susceptible (S) and -resistant *C. jejuni* (R), the transcriptional profile and relative *in vitro* and *in vivo* phenotype determination were implemented to decipher the function and regulation mechanism of *Cj0440c.*

## Materials and Methods

### Plasmids, Bacterial Strains, and Growth Conditions

The *C. jejuni* NCTC11168 (designated as **S**) was kindly provided by Chinese Center for Disease Control and Prevention. *C. jejuni* strains were routinely cultured in Mueller-Hinton (MH) medium at 42°C under microaerobic conditions (5% O_2_, 10% CO_2_, and 85% N_2_) in the anaerobic incubator (YQX-II, Shanghai, China) (Mace et al., 2015). The *Escherichia coli* DH5α was grown aerobically in Luria-Bertani medium at 37°C. The erythromycin-resistant *C. jejuni* strain 68-ER (designated as **R**) was descendant of *C. jejuni* NCTC11168 resulting from *in vitro* step-wise selection by erythromycin. Plasmids pGEM-T (Promega, Madison, WI, USA) and pMW10 was kindly provided by China Agricultural University and used for mutant vector construction.

### Construction of Isogenic Δ*Cj0440c* Mutants

The DNA fragment containing *Cj0440c* gene and its flanking regions was amplified from *C. jejuni* NCTC 11168 genome using *Pfu* polymerase (Promega) with primers of Cj0440cF_2_ and Cj0440cR_2_ (**Table 1**) and was cloned into pGEM-T easy vector (Promega,) to generate plasmid pCJ0440c. Primers pCj0440cU and pCj0440cL (**Table 1**) carrying endonuclease restriction sites of KpnI and XbaI were used to inversely amplify DNA fragment from the vector of pCJ0440c using Taq and Pfu polymerase (8:1). A kanamycin resistance cassette (*kan*) was amplified from pMW10 plasmid with Pfu polymerase (Promega) using primers KanF and KanR (**Table 1**) which have the same restriction sites of KpnI and XbaI. The amplified DNA fragments of inverse PCR and *kan* were digested with KpnI and XbaI and purified with a PCR clean-up kit (Generay, Shanghai, China). The digested inverse PCR product was ligated to the *kan* cassette using T4 DNA ligase (Takara, Dalian, China) to obtain the construct plasmid pCJ0440c-Kan, which was then transformed into *E. coli* DH5α. The purified plasmid of pCJ0440c-Kan was introduced into S and R via electroporation according to the method described previously (Jeon et al., 2011). Insertional mutants, named SM and RM, respectively, were selected on MH agar plates with 25 μg/ml kanamycin and 50 μg/ml ampicillin. Both PCR and sequencing analysis of the *Cj0440c* mutants (SM and RM) confirmed that the mutation resulted in deletion of 200 bp of coding sequence in *Cj0440c* and simultaneous insertion of the *kan* gene into the same location.

**Table 1:** Primers used for construction of *Cj0440c* mutant and for real-time qPCR.

Primer name	Primer Sequence (from 5′ to 3′)	Productsize (bp)
Primers used for construction and confirmation of *Cj0440c* mutant		
Cj0440c-F2	AATACCAGAAGCCGAAAC	2315
Cj0440c-R2	GAGGGTGAAATAGAAGGG	
pCj0440c-U	GGGGTACCAGATCATCCTTACAAGGAAT *Kpn*I site	5100
pCj0440c-L	GCTCTAGATTCATAGCAAAACGAAGT *Xba*I site	
Kan-F	GCTCTAGAAATGGGCAAAGCAT *Xba*I site	1203
Kan-R	GGGGTACCATAATGCTAAGACAAT *Kpn*I site	
Primers used for real-time qPCR		
Cj1339cF	TCCATTAAACGGTTGATATCTGCTT	125
Cj1339cR	AAGGCTATGGATGAGCAACTTAAAAT	
Cj1328F	CTTTTAGCGATGCTTTTGAAGACTTA	126
Cj1328R	CGCCACATAAATGCACTAAAGG	
Cj1294F	GGCGTAAAACACGCTTGTGTATT	79
Cj1294R	TTTCTTGGACACCTAGTGCTGTGTA	
Cj1338cF	TTACCATTGTTGATAGCTTGACCTAAA	75
Cj1338cR	TGCTTCAGGGATGGCGATA	
Cj0043F	GGGTTCTCCTGTTGCAAGTGA	75
Cj0043R	GCCCCTAAAACCCCAAAAAAT	
Cj0697F	TGGTTCAGACCAAAGATGGA	138
Cj0697R	TGCCAGCATTCTGAGGATTA	
Cj1242F	AAGACATTGATCTTGGTGCTG	143
Cj1242R	ATTGTTTGTGGCATTTCCTG	
Cj1385F	GGAAACTGGGACTTGGTAGGAA	83
Cj1385R	TGAGTATGGATGAAATCAGGGAATT	
Cj1464F	CGAGTAAAATCGCAGAGCAG	69
Cj1464R	TCGCAGCAGCTGTAGCTTT	
q16SF	GCTCGTGTCGTGAGATGTTG	199
q16SR	GCGGTATTGCGTCTCATTGTAT	

### RNA Microarray and Data Analysis

The transcriptional difference between *Cj0440c* mutants and their parental strains (SM&S and RM&R) was examined by microarray (CapitalBio Corporation, Shanghai, China). Briefly, the strains were separately grown in MH broth for 24 h at 42°C under microaerophilic conditions with shaking. Immediately after the incubation, twice volume of RNA protective reagent (Qiagen, Germantown, MD, USA) was added to the culture (with same OD_600_ of 0.3) to stabilize mRNA. The total RNA from each sample was extracted using RNeasy Protect Mini Kit (Qiagen) and purified using Nucleo^®^Spin RNA clean-up kit (Macherey-nagel, Germany). The RNA quality and quantity was determined by formaldehyde denatured gel electrophoresis and Nanodrop^TM^ 2000 Spectrophotometer (Thermo Fisher Scientific, Waltham, MA, USA). The cDNA was synthesized from the extracted RNA using iScript cDNA synthesis kit (Bio-Rad, Hercules, CA, USA). The cDNA was labeled by Cy5 or Cy3 dye and co-hybridized onto one microarray slide (NimbleGen 4 K × 72K), scanned by Axon Instruments Gene Pix 4000B (Union City, CA, USA) and read by Gene Pix Pro 6.0 (Axon Instruments). Microarray data were analyzed using Array Star software. The genes with False Discovery Rate (FDR)-corrected *q*-values < 0.01 and fold change >2 were selected as differentially expressed genes and subjected to NCBI gene annotation, KEGG pathway analysis and STRING 9.05 protein network analysis.

### Microarray Data Accession Number

The microarray data obtained in this study have been deposited in the NCBI Gene Expression Omnibus database^1^ and assigned accession number GSE49255 and GSE49256.

### qRT-PCR

The same batches cDNA of *Cj0440c* mutants (SM and RM) and their parental strains (S and R) used in microarray were subjected to qRT-PCR analysis to confirm the transcriptional difference of some respective genes identified by microarray following method described in previous study (Hao et al., 2010). Briefly, the PCR amplification was performed in IQ5 Multicolor Real-time PCR Detection System (Bio-Rad). The cycling conditions were as follows: 3 min of pre-incubation at 95°C, followed by 30 cycles of 10 s at 95°C and 40 s at 60°C. The primer sets used for specific genes are listed in **Table 1**. 16S rDNA was used as an internal control for normalization. The experiment was done in triplicate to obtain the average value of fold change. The student’s *t*-test was performed to analyze the significant difference between mutants and their parental stains.

### Antimicrobial Susceptibility Test

Minimum inhibitory concentrations (MICs) of nine antimicrobial agents (azithromycin, erythromycin, tylosin, ciprofloxacin, olaquindox, chloromycetin, tetracycline, gentamicin, and ceftriaxone) were determined using agar dilution method recommended by Clinical and Laboratory Standards Institute (CLSI). *C. jejuni* ATCC 33560 was used as a quality control strain.

### Transmission Electron Microscopy

The presence and length of flagella on the four *C. jejuni* strains (S, SM, R and RM) were examined using transmission electron microscopy according to a previously described method (Barrero-Tobon and Hendrixson, 2014; Matsunami et al., 2016). Briefly, bacterial suspensions were obtained after washing plate with 2 ml sterile phosphate-buffered saline and spotted on carbon-coated copper grids. The cells were stained with 2% phosphotungstic acid (pH 6.7) for 1 min. Samples were observed employing a HITACHI H-7650 transmission electron microscope (Hitachi, Japan).

### *In Vitro* Growth Determination

To compare the growth kinetics of the mutants with that of the parental strains, a fresh culture (100 μL) of each *C. jejuni* strain (0.5 McFarland) was inoculated into 100 mL MH broth and the cultures were incubated at 42°C under microaerobic conditions for 36 h with shaking. The growth kinetics was determined by measuring the absorbance in 600 nm (OD_600_) at 0, 4, 8, 12, 20, 22, 24, 27, 31, 33, and 36 h post-inoculation.

### Single and Competitive Colonization in Chicken

Newly hatched broiler chickens (White Leghorns) were purchased from Zhengda Limited Company (Wuhan, China). All the broiler chickens were examined for *C. jejuni* to ascertain that birds are free of *C. jejuni* prior to infection all the chickens (Hao et al., 2015).

These chickens were randomly assigned to seven groups with 6 to 10 chickens per group. One group was used as a control. Four groups were used for single colonization test in which 10^9^ CFU *C. jejuni* strains (S, SM, R and RM) were individually inoculated via oral gavage into each group. Another two groups were used for pairwise competition test in which 10^9^ CFU *C. jejuni* pairwise mixtures (S&SM or R&RM) were inoculated via oral gavage to each group. Fecal samples were collected from each bird at 3, 6, 9, and 12 days’ post-infection. The CFU of S, SM, R and RM were determined using *Campylobacter* selective CCDA agar (Oxoid, Thermo Fisher Scientific, Waltham, MA, USA) with or without 25 μg/ml Kanamycin or 50 μg/ml erythromycin. Each sample was spread onto three respective selective plates to obtain the average CFU.

The significance of differences between mutant and parental strain in colonization at each sampling time point was determined by using Student’s *t*-test, Welch’s *t*-test to allow for non-constant variation across treatment groups, and the Wilcoxon rank-sum test to allow for non-normality (Guo et al., 2008; Luangtongkum et al., 2012; Xia et al., 2013). Differences were considered significant at a *P*-value of <0.01.

### Ethics Statement

The animal study was approved by Animal Ethics Committee of Huazhong Agricultural University (HZAUCH 2013-002) and the Animal Care Center, Hubei Science and Technology Agency in China (SYXK 2013-0044). All experimental procedures in this study were performed according to the guidelines of the committee on the use and care of the laboratory animals in Hubei Province, China. All the animals were monitored throughout the study for any signs of adverse effects.

## Results

### Differentially Expressed Genes in SM and RM

**FIGURE 1:**
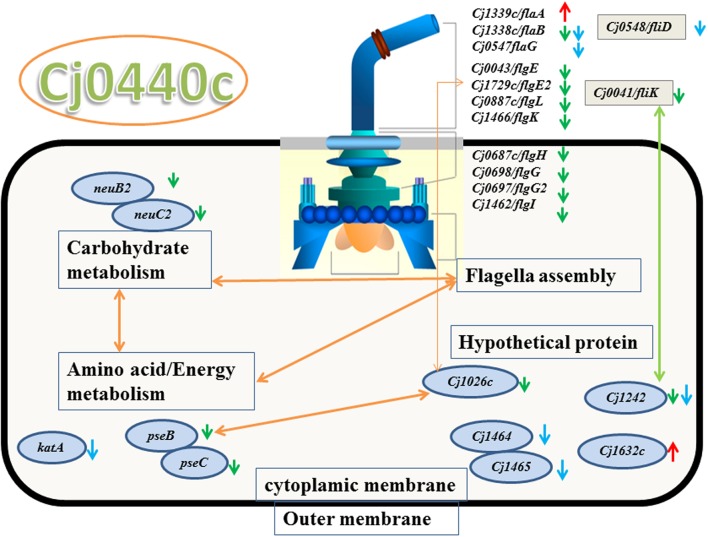
**Significant genes in *Cj0440c*-inactivated mutants and their relationship based on KEGG and STRING protein network analysis**. Green arrow and red arrow were genes down-regulated and up-regulated in SM, respectively. Genes with blue arrow were down-regulated in RM. The yellow double sided arrow means positive relationship between these genes and green double sided arrow means negative relationship between these genes.

The target gene *Cj0440c* was down-regulated in the *Cj0440c*-inactivated mutants (SM and RM). The other differentially expressed genes in *Cj0440c* mutants (SM and RM) compared to their parental strains (S and R) were shown in **Tables 2**, **3**. The relationship of these different genes was summarized in **Figure 1**.

**Table 2:** Transcriptional difference in the mutant SM comparing to its parental strain S determined by microarray.

Function class	Gene name	Gene function	Fold change
Target gene	*Cj0440c*	Putative transcriptional regulator	-24.3
Cell motility/signal transduction	*Cj1339c/flaA*	Flagellin	2.1
	*Cj1338c/flaB*	Flagellin	-3.5
	*Cj1729c/flgE2*	Flagellar hook protein FlgE	-2.2
	*Cj0887c/flgL*	Flagellar hook-associated protein FlgL	-2.4
	*Cj1466/flgK*	Flagellar hook-associated protein FlgK	-3.3
	*Cj0043/flgE*	Flagellar hook protein	-3.5
	*Cj1462/flgI*	Flagellar basal body P-ring protein	-3.0
	*Cj0698/flgG*	Flagellar basal body rod protein FlgG	-3.2
	*Cj0687c/flgH*	Flagellar basal body L-ring protein	-3.2
	*Cj0697/flgG2*	Flagellar basal-body rod protein	-3.6
	*Cj0041/fliK*	Putative flagellar hook-length control protein	-4.5
Carbohydrate metabolism	*Cj1327/neuB2*	*N*-acetylneuraminic acid synthetase	-3.1
	*Cj1328/neuC2*	UDP-*N*-acetylglucosamine 2-epimerase	-2.4
Amino acid/energy metabolism	*Cj1293/pseB*	UDP-GlcNAc-specific C4,6 dehydratase/C5 epimerase	-2.2
	*Cj1294/pseC*	C4 aminotransferase specific for PseB product	-2.5
Hypothetical proteins	*Cj1026c*	Putative lipoprotein	-2.1
	*Cj1242*	Hypothetical protein	-2.3
	*Cj1632c*	Putative periplasmic protein	5.7

*Only flaA and Cj1632c were up-regulated*.

**Table 3:** Transcriptional difference in the mutant RM comparing to its parental strain R determined by microarray.

Function class	Gene name	Gene function	Foldchange
Target gene	*Cj0440c*	Putative transcriptional regulator	-35.2
Cell motility/signal transduction	*Cj1338c/flaB*	Flagellin	-2.3
	*Cj0547/flaG*	Flagellar protein FlaG	-2.3
	*Cj0548/fliD*	Flagellar capping protein	-2.1
	*Cj0042/flgD*	Flagellar basal body rod modification protein	-2.7
Energy metabolism	*Cj1385/katA*	Catalase	-2.5
Hypothetical proteins	*Cj1464/flgM*	Hypothetical protein	-4.4
	*Cj1465*	Hypothetical protein	-3.8
	*Cj1242*	Hypothetical protein	-2.0
	*Cj0391c*	Hypothetical protein	-2.1

A flagellin gene (*flaA*) and a gene (*Cj1632c*) encoding a putative periplasmic protein were up-regulated in SM as compared to S (indicated by red arrows in **Figure 1**). Among the down-regulated genes in SM (indicated by green arrows in **Figure 1**), 10 genes (*flaB*, *flgE*, *flgE2*, *flgG*, *flgG2*, *flgH*, *flgI*, *flgK*, *flgL*, and *fliK*) are possible involve in flagellar assembly; 2 genes (*pseB* and *pseC*) in carbohydrate metabolism; 2 genes (*neuB_2_* and *neuC*) in surface glycoprotein metabolism.

None of the genes were up-regulated genes were found in RM when compared the expression of different genes with R. Eleven down regulated genes in RM (indicated by blue arrows in **Figure 1**) are flagellar associated genes (*flgD*, *fliD*, *flaG* and *flaB*), a catalase encoding gene (*katA*) and four genes with unknown function (*flgM*, *Cj1465*, *Cj0391*, and *Cj1242*).

When submitted to STRING 9.05 and KEGG pathway analysis, the result showed that 10 flagellar genes were interacted with other down-regulated genes (*pseB*/*C*, *neuB_2_*/*C2* and *Cj1026c*) (**Figure 1**).

**FIGURE 2:**
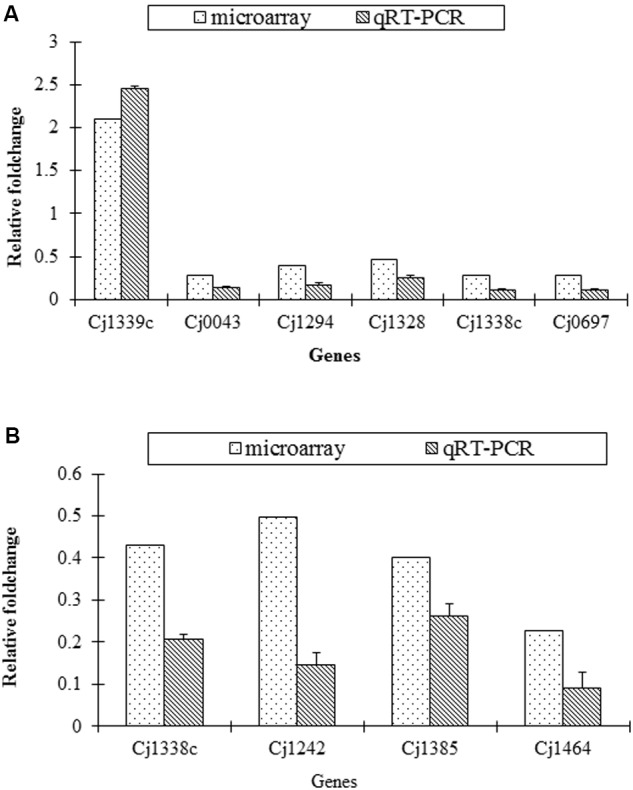
**Confirmation of transcriptional change of target genes in the mutants and the parental strains by qRT-PCR. (A)** Fold change of six genes in SM versus S; **(B)** Fold change of four genes in RM versus R.

The transcriptional change of several representative genes detected in microarray was validated by qRT-PCR. The similar change of the selected genes was found both in microarray and qRT-PCR (**Figure 2**).

The microarray data obtained in this study have been deposited in the NCBI Gene Expression Omnibus database^2^ and assigned accession numbers GSE49255 (RM&R) and GSE49255(SM&S).

### Antimicrobial Susceptibility of *Cj0440c* Mutants

As shown in **Table 4**, there was no significant difference between MIC of nine antimicrobial agents in *Cj0440c* mutants (SM and RM) comparing to their parental strains (S and R). Inactivation of *Cj0440c* did not affect antimicrobial susceptibility of *C. jejuni*.

**Table 4:** Minimum inhibitory concentration (MIC) of *Cj0440c* mutant strains and parental strains to different drugs.

Strains	MIC to different drugs (μg/mL)								
	ERY	TYL	AZI	TET	CIP	CHL	GEN	CRO	OLA
S	1	4	0.0625	0.5	0.125	2	0.5	16	2
SM	1	4	0.0625	0.5	0.125	2	0.5	16	1
R	256	256	32	0.5	0.125	2	0.5	16	2
RM	256	256	32	0.5	0.125	2	0.5	16	1

*S was *C. jejuni* NCTC 11168; SM was Cj0440c deletion mutant of S; R was Erythromycin resistant strain selected from *C. jejuni* NCTC 11168; RM was Cj0440c deletion mutant of R. The drugs included erythromycin (ERY), tylosin (TYL), azithromycin (AZM), tetracydine (TET), ciprofloxacin (CIP), chloramphenicol (CHL), gentamicin (GEN), ceftriaxone (CRO) and olaquindox (OLA)*.

### Flagella Presence and Length

**FIGURE 3:**
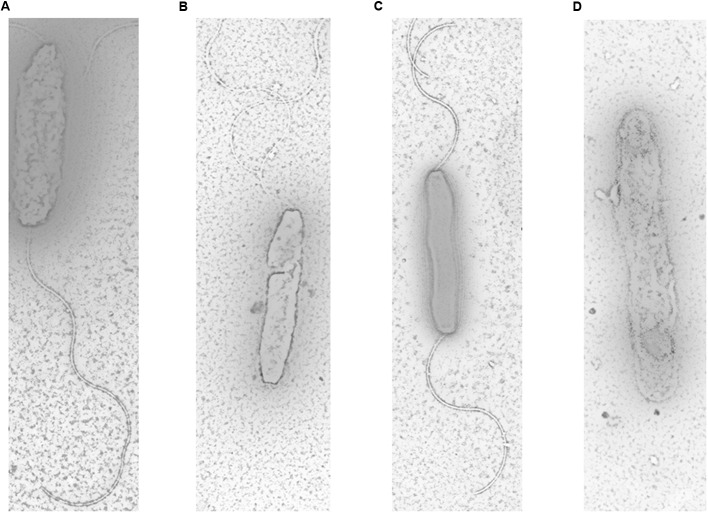
**Flagella morphology of S** **(A)**, SM **(B)**, R **(C)**, and RM **(D)** under transmission electron microscope. The magnification used for TEM images in the captionis are 1 μm.

The electron micrographs of all tested strains were shown in **Figure 3**. The results showed that parental strains (S and R) had long, spiral and complete flagella filaments in two sides (**Figures 3A,C**). However, SM had shorter filaments in only one side (**Figure 3B**). No filaments of RM were detected in RM (**Figure 3D**). These findings indicated that *Cj0440c* may affect the formation of flagella in *C. jejuni.*

### *In Vitro* Growth of *Cj0440c* Mutants

**FIGURE 4:**
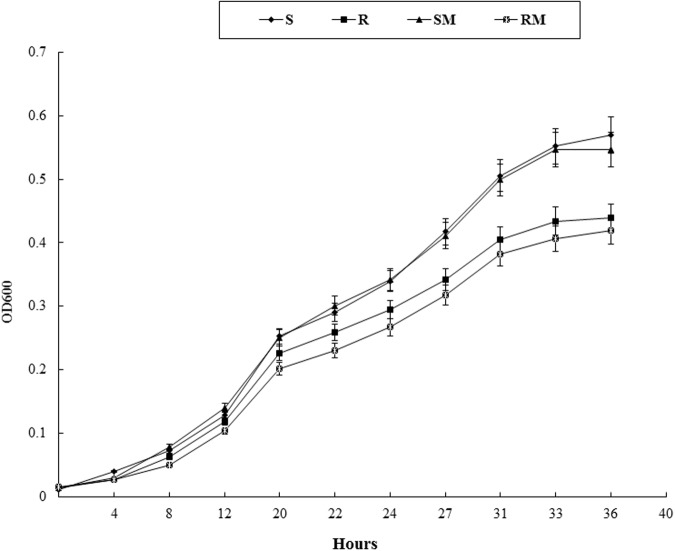
**The growth curve of *Cj0440c* mutants (SM and RM) and their parental strains (S and R)**.

Growth kinetics of *Cj0440c* mutants (SM and RM) and their parental strains (S and R) were determined in MH broth. No significant difference in growth rate was observed between SM and S. The RM exhibited slower growth rate compared to its parental R, however, the difference was not significant (**Figure 4**).

### *In Vivo* Colonization of *Cj0440c* Mutants

**FIGURE 5:**
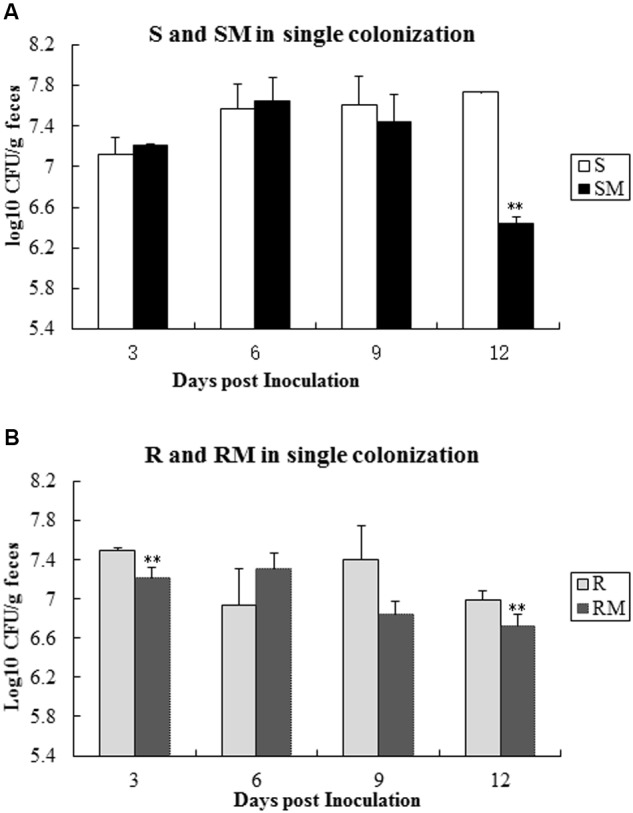
**Single colonization of four *C. jejuni* strains in chickens after oral inoculation. (A)** Colonization of S and SM; **(B)** Colonization of R and RM. The asterisk (^∗∗^) represent statistically significant difference with *P* ≤ 0.01 comparing with parental strains, respectively.

To determine the colonization capacity, broiler chickens were infected individually with four *C. jejuni* strains (S, R, SM and RM). All the strains were able to colonize in chicken intestinal tract, albeit at different rate. Comparing with the parental strains (S or R), the *Cj0440c* mutants (SM and RM) showed a significant reduction in colonization on 12 days’ post-inoculation (**Figure 5**).

**FIGURE 6:**
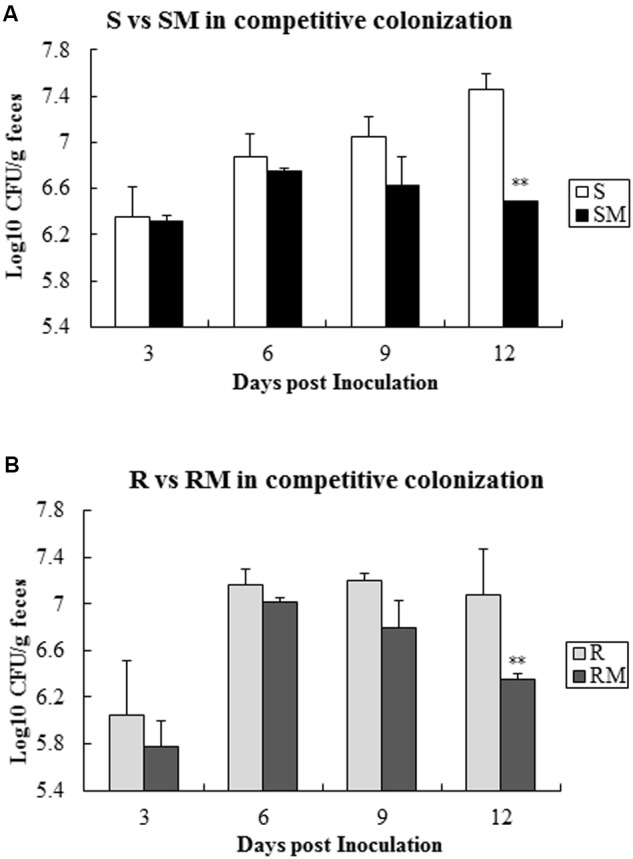
**Competitive colonization of *Cj0440c* mutants with their patent strains (SM&S and RM&R). (A)** Competitive colonization of the pair of SM&S; **(B)** Competitive colonization of the pair of RM&R. The asterisk (^∗∗^) represent statistically significant difference with *P* ≤ 0.01 comparing with parental strains, respectively.

When the two pairs of *C. jejuni* strains (SM&S and RM&R) were infected chickens with one pair at a time, *Cj0440c* mutants (SM and RM) exhibited lower colonization level compared to their parental strains (S and R) after 9 days’ post-inoculation (**Figure 6**).

## Discussion

*Campylobacter jejuni* is a very common foodborne pathogen in the developed world. Its biology and pathogenicity is largely unknown (Young et al., 2007). *Cj0440c* is a putative transcriptional regulator and an increased transcriptional level expression was detected in the erythromycin-resistant *C. jejuni* (Hao et al., 2013). The gene may encode a TENA/THI-4/PQQC family protein. TENA is one of a number of proteins that enhance the expression of extracellular enzymes (e.g., alkaline protease, neutral protease and levansucrase) (Pang et al., 1991). The extracellular enzymes may be regulated by the master regulator of flagellar genes (e.g., *flhDC*) (Cui et al., 2008). THI-4 protein is involved in thiamine biosynthesis (Akiyama and Nakashima, 1996). This family also includes bacterial coenzyme pyrroloquinoline quinone (PQQ) synthesis protein C (PQQC) proteins. PQQ is the prosthetic group of several bacterial enzymes, including methanol dehydrogenase of methylotrophs and the glucose dehydrogenase (Toyama et al., 2002, 2007). In *E. coli*, PQQ biosynthesis may be affected by *tldD* gene which encodes a peptidase involved in processing of small peptides (Holscher and Gorisch, 2006). The *tldD* may lead to chromosomal DNA relaxation and subsequent derepression of *cdtB* and *lgeR* which may regulate the expression of some flagellar genes (Haghjoo and Galan, 2007). Therefore, the TENA/THI-4/PQQC family may have some indirect relationship with flagellar genes.

The flagella formation plays an important role in the pathogenesis of *Campylobacter* including motility, microcolony formation, biofilm formation, autoagglutination, protein secretion, adherence to host cell, and host invasion (Guerry et al., 2006; Guerry, 2007). The major groups of down-regulated genes in *Cj0440c* mutants (SM and RM) were involved in flagellar assembly, including 11 genes (*flaB*, *flgE*/*E2*/L/K/H/*G*/*G2*/*I*, *flgK*, *flgL*, *fliK*) in SM and 4 genes (*flaB/G*, *flgD*, *fliD*) in RM (**Figure 1**). The down-regulation of those flagella-associated genes in *Cj0440c* mutants can reasonably explain why SM and RM lose one or two sides of filament. The reduced colonization of *Cj0440c* mutants may result from the down-regulation of flagella genes.

A second group of genes (*pseB, pseC, neuB2* and *neuC2*) down-regulated in SM were involved in *O*-linked glycosylation which was also essential for flagellin assembly. The *pseB/C* in *C. jejuni* contribute in glycosylation modifications of flagellin, often by non-specifically modifying the surface-exposed Thr, Ser, and Tyr residues of filament proteins FlaA and FlaB (Ewing et al., 2009). While *neuB2/C2* requires in *O*-linked glycosylation which may contribute to flagella antigen diversity of *Campylobacter* (Linton et al., 2000; Sundaram et al., 2004; Tabei et al., 2009). The down-regulation of these glycosylation-associated genes in SM suggested that *Cj0440c* may play an important role in flagella assembly.

Several hypothetical genes (*Cj1026c, Cj1242, Cj1464, Cj1465* and *Cj0391c*) were down-regulated in SM or RM. The Cj1026c (FlgP) was essential for motility of *C. jejuni* and possible encode the promoter of *flaG* (Sommerlad and Hendrixson, 2007). The Cj1464 (FlgM) may regulate temperature-dependent FlgM/FliA complex formation and flagella length of *C. jejuni* (Wösten et al., 2010). The *Cj0391c* generally co-expressed with flagella-associated genes and involved in biofilm formation of *Campylobacter* (Kalmokoff et al., 2006). The down-regulation of these genes suggested that *Cj0440c* may be closely associated with flagella biosynthesis and assembly.

All our data showed that *Cj0440c* may have close relationship with flagella biosynthesis and assembly, however, the precise role of *Cj0440c* in flagella formation pathway is yet to be determined. Flagellar biogenesis in *C. jejuni* requires three distinct sigma factors, including σ^80^, σ^54^ (or RpoN) and σ^28^ (or FliA) (McCarter, 2006; Anderson et al., 2010). The FlgS/FlgR two-component system is required for transcription of the RpoN regulon (Joslin and Hendrixson, 2009). The FliK likely part of a negative feedback loop that turns off expression of σ^54^-dependent genes (Ryan et al., 2005; Kamal et al., 2007). The FlgM (anti σ^28^) may negatively regulate the class III motility genes (Wang et al., 2005). The present study showed that the transcription of *fliK* was down-regulated in SM and the transcription of *flgM* (*Cj1464*) was down-regulated in RM. The down-regulation of *fliK* and *flgM* can influence the down-regulation of class II and class III motility. The roles of *Cj0440c* on flagellar genes are complex and further investigations are required.

The transcriptional change of majority parts of the genes was similar in both SM and RM except for few differences. The *flaA* and *Cj1632c* were up-regulated and *O*-linked glycosylation was down-regulated only in SM, while *katA*, encoding a sole catalase, was down-regulated in RM but not in SM. The flagellar filaments of *Campylobacter* spp. were composed primarily by FlaA and FlaB flagellin (Guerry et al., 1991). The *flaA* was merely up-regulated in SM but *flaB* was down-regulated in both SM and RM. Findings of our study suggested that that the role *Cj0440c* on transcription of FlaA and FlaB flagellin are different in Ery^s^ and in Ery^r^ *C. jejuni*. The *katA* involves in oxidative stress and ROS defense which was essential for intra-macrophage persistence and environmental stress survival of *Campylobacter* (Farr and Kogoma, 1991; Day et al., 2000; Vliet et al., 2002; Flint et al., 2012). The down-regulation of *katA* in RM suggested that *Cj0440c* may interact with *katA* to improve their survival capacity in environmental stress.

The macrolide-resistance in *C. jejuni* generally suffered a fitness cost, however, several other factors may compensate the adaptation weakness (Björkman and Andersson, 2000; Kugelberg et al., 2006; Nilsson et al., 2006; Hao et al., 2010, 2013; Luangtongkum et al., 2012). Our previous study showed that *Cj0440c* was over-expressed in the Ery^r^ *C. jejuni* (Hao et al., 2013). The result from the present study suggests that *Cj0440c* plays a role in compensating the fitness cost of erythromycin resistance through the positive relationship with flagellar and other related genes.

## Conclusion

*Cj0440c* regulates expression of genes involved in flagella biosynthesis and assembly which consequently affects the growth or colonization of *C. jejuni in vitro* and *in vivo* environment.

## Author Contributions

Conceived and designed the experiments: XF, HH, YW, XW, and ZY. Performed the experiments: XF and HH. Analyzed the data: XF, HH, JH, SF, GC, LH, and ZY. Contributed reagents/materials/analysis tools: ZY, ZL, MD, and HH. Wrote the paper: HH, XF, JH, SF, and ZY.

## Conflict of Interest Statement

The authors declare that the research was conducted in the absence of any commercial or financial relationships that could be construed as a potential conflict of interest.

## Footnotes

1 http://www.ncbi.nlm.nih.gov/geo/database

2 https://www.ncbi.nlm.nih.gov/geo/

## References

[B1] Akiyama M. Nakashima H. (1996). Molecular cloning of thi-4, a gene necessary for the biosynthesis of thiamine in *Neurospora crassa*. *Curr. Genet.* 30 62–67. 10.1007/s002940050101 8662211

[B2] Anderson J. K. Smith T. G. Hoover T. R. (2010). Sense and sensibility: flagellum-mediated gene regulation. *Trends Microbiol.* 18 30–37. 10.1016/j.tim.2009.11.001 19942438 PMC2818477

[B3] Barrero-Tobon A. M. Hendrixson D. R. (2014). Flagellar biosynthesis exerts temporal regulation of secretion of specific *Campylobacter jejuni* colonization and virulence determinants. *Mol. Microbiol.* 93 957–974. 10.1111/mmi.12711 25041103 PMC4150830

[B4] Björkman J. Andersson D. I. (2000). The cost of antibiotic resistance from a bacterial perspective. *Drug Resist. Updat.* 3 237–245. 10.1054/drup.2000.0147 11498391

[B5] Byers D. M. Gong H. (2007). Acyl carrier protein: structure-function relationships in a conserved multifunctional protein family. *Biochem. Cell Biol.* 85 649–662. 10.1139/O07-109 18059524

[B6] Cui Y. Chatterjee A. Yang H. Chatterjee A. K. (2008). Regulatory network controlling extracellular proteins in *Erwinia carotovora* subsp. carotovora: FlhDC, the master regulator of flagellar genes, activates *rsmB* regulatory RNA production by affecting gacA and hexA (*lrhA*) expression. *J. Bacteriol.* 190 4610–4623. 10.1128/JB.01828-07 18441056 PMC2446818

[B7] Day W. A. Sajecki J. L. Pitts T. M. Joens L. A. (2000). Role of catalase in *Campylobacter jejuni* intracellular survival. *Infect. Immun.* 68 6337–6345. 10.1128/IAI.68.11.6337-6345.2000 11035743 PMC97717

[B8] ECDC EFSA EMEA and SCENIHR (2009). Joint opinion on antimicrobial resistance (AMR) focused on zoonotic infections. *EFSA J.* 7 1372.

[B9] Epps S. V. Harvey R. B. Hume M. E. Phillips T. D. Anderson R. C. Nisbet D. J. (2013). Foodborne *Campylobacter*: infections, metabolism, pathogenesis and reservoirs. *Int. J. Environ. Res. Public Health* 10 6292–6304. 10.3390/ijerph10126292 24287853 PMC3881114

[B10] Ewing C. P. Andreishcheva E. Guerry P. (2009). Functional characterization of flagellin glycosylation in *Campylobacter jejuni* 81-176. *J. Bacteriol.* 191 7086–7093. 10.1128/JB.00378-09 19749047 PMC2772469

[B11] Farr S. B. Kogoma T. (1991). Oxidative stress responses in *Escherichia coli* and *Salmonella typhimurium*. *Microbiol. Rev.* 55 561–585. 1779927 10.1128/mr.55.4.561-585.1991 PMC372838

[B12] Flint A. Sun Y.-Q. Stintzi A. (2012). *Cj1386* is an ankyrin-containing protein involved in heme trafficking to catalase in *Campylobacter jejuni*. *J. Bacteriol.* 194 334–345. 10.1128/JB.05740-11 22081390 PMC3256678

[B13] Gibreel A. Kos V. N. Keelan M. Trieber C. A. Levesque S. Michaud S. (2005). Macrolide resistance in *Campylobacter jejuni* and *Campylobacter coli*: molecular mechanism and stability of the resistance phenotype. *Antimicrob. Agents Chemother.* 49 2753–2759. 10.1128/AAC.49.7.2753-2759.2005 15980346 PMC1168676

[B14] Gibreel A. Taylor D. E. (2006). Macrolide resistance in *Campylobacter jejuni* and *Campylobacter coli*. *J. Antimicrob. Chemother.* 58 243–255. 10.1093/jac/dkl210 16735431

[B15] Guerry P. (2007). *Campylobacter* flagella: not just for motility. *Trends Microbiol.* 15 456–461. 10.1016/j.tim.2007.09.006 17920274

[B16] Guerry P. Alm R. Power M. Logan S. Trust T. (1991). Role of two flagellin genes in Campylobacter motility. *J. Bacteriol.* 173 4757–4764. 10.1128/jb.173.15.4757-4764.1991 1856171 PMC208154

[B17] Guerry P. Ewing C. P. Schirm M. Lorenzo M. Kelly J. Pattarini D. (2006). Changes in flagellin glycosylation affect *Campylobacter* autoagglutination and virulence. *Mol. Microbiol.* 60 299–311. 10.1111/j.1365-2958.2006.05100.x 16573682 PMC1424674

[B18] Guo B. Wang Y. Shi F. Barton Y. W. Plummer P. Reynolds D. L. (2008). CmeR functions as a pleiotropic regulator and is required for optimal colonization of *Campylobacter jejuni* in vivo. *J. Bacteriol.* 190 1879–1890. 10.1128/JB.01796-07 18178742 PMC2258875

[B19] Haghjoo E. Galan J. E. (2007). Identification of a transcriptional regulator that controls intracellular gene expression in *Salmonella* Typhi. *Mol. Microbiol.* 64 1549–1561. 10.1111/j.1365-2958.2007.05754.x 17555437

[B20] Hao H. Dai M. Wang Y. Chen D. Yuan Z. (2010). Quantification of mutated alleles of 23S rRNA in macrolide-resistant *Campylobacter* by TaqMan real-time polymerase chain reaction. *Foodborne Pathog. Dis.* 7 43–49. 10.1089/fpd.2009.0339 19743927

[B21] Hao H. Liu J. Kuang X. Dai M. Cheng G. Wang X. (2015). Identification of *Campylobacter jejuni* and determination of point mutations associated with macrolide resistance using a multiplex TaqMan MGB real-time PCR. *J. Appl. Microbiol.* 118 1418–1425. 10.1111/jam.12793 25766481

[B22] Hao H. Yuan Z. Shen Z. Han J. Sahin O. Liu P. (2013). Mutational and transcriptomic changes involved in the development of macrolide resistance in *Campylobacter jejuni*. *Antimicrob. Agents Chemother.* 57 1369–1378. 10.1128/AAC.01927-12 23274667 PMC3591901

[B23] Holscher T. Gorisch H. (2006). Knockout and overexpression of pyrroloquinoline quinone biosynthetic genes in *Gluconobacter oxydans* 621H. *J. Bacteriol.* 188 7668–7676. 10.1128/JB.01009-06 16936032 PMC1636293

[B24] Hughes R. A. Cornblath D. R. (2005). Guillain-Barré syndrome. *Lancet* 366 1653–1666. 10.1016/S0140-6736(05)67665-9 16271648

[B25] Jeon B. Wang Y. Hao H. Barton Y.-W. Zhang Q. (2011). Contribution of CmeG to antibiotic and oxidative stress resistance in *Campylobacter jejuni*. *J. Antimicrob. Chemother.* 66 79–85. 10.1093/jac/dkq418 21081547 PMC3001851

[B26] Joslin S. N. Hendrixson D. R. (2009). Activation of the *Campylobacter jejuni* FlgSR two-component system is linked to the flagellar export apparatus. *J. Bacteriol.* 191 2656–2667. 10.1128/JB.01689-08 19201799 PMC2668382

[B27] Kalmokoff M. Lanthier P. Tremblay T.-L. Foss M. Lau P. C. Sanders G. (2006). Proteomic analysis of *Campylobacter jejuni* 11168 biofilms reveals a role for the motility complex in biofilm formation. *J. Bacteriol.* 188 4312–4320. 10.1128/JB.01975-05 16740937 PMC1482957

[B28] Kamal N. Dorrell N. Jagannathan A. Turner S. M. Constantinidou C. Studholme D. J. (2007). Deletion of a previously uncharacterized flagellar-hook-length control gene fliK modulates the sigma54-dependent regulon in *Campylobacter jejuni*. *Microbiology* 153 3099–3111. 10.1099/mic.0.2007/007401-0 17768253

[B29] Kassem I. I. Khatri M. Esseili M. A. Sanad Y. M. Saif Y. M. Olson J. W. (2012). Respiratory proteins contribute differentially to *Campylobacter jejuni’*s survival and *in vitro* interaction with hosts’ intestinal cells. *BMC Microbiol.* 12:258 10.1186/1471-2180-12-258 PMC3541246 23148765

[B30] Kugelberg E. Kofoid E. Reams A. B. Andersson D. I. Roth J. R. (2006). Multiple pathways of selected gene amplification during adaptive mutation. *Proc. Natl. Acad. Sci. U.S.A.* 103 17319–17324. 10.1073/pnas.0608309103 17082307 PMC1633709

[B31] Linton D. Karlyshev A. V. Hitchen P. G. Morris H. R. Dell A. Gregson N. A. (2000). Multiple N-acetyl neuraminic acid synthetase (neuB) genes in *Campylobacter jejuni*: identification and characterization of the gene involved in sialylation of lipo-oligosaccharide. *Mol. Microbiol.* 35 1120–1134. 10.1046/j.1365-2958.2000.01780.x 10712693

[B32] Luangtongkum T. Shen Z. Seng V. W. Sahin O. Jeon B. Liu P. (2012). Impaired fitness and transmission of macrolide-resistant *Campylobacter jejuni* in its natural host. *Antimicrob. Agents Chemother.* 56 1300–1308. 10.1128/AAC.05516-11 22183170 PMC3294946

[B33] Mace S. Haddad N. Zagorec M. Tresse O. (2015). Influence of measurement and control of microaerobic gaseous atmospheres in methods for *Campylobacter* growth studies. *Food Microbiol.* 52 169–176. 10.1016/j.fm.2015.07.014 26338132

[B34] Matsunami H. Barker C. S. Yoon Y. H. Wolf M. Samatey F. A. (2016). Complete structure of the bacterial flagellar hook reveals extensive set of stabilizing interactions. *Nat. Commun.* 7:13425 10.1038/ncomms13425 PMC5097172 27811912

[B35] McCarter L. L. (2006). Regulation of flagella. *Curr. Opin. Microbiol.* 9 180–186. 10.1016/j.mib.2006.02.001 16487743

[B36] Nilsson A. I. Zorzet A. Kanth A. Dahlström S. Berg O. G. Andersson D. I. (2006). Reducing the fitness cost of antibiotic resistance by amplification of initiator tRNA genes. *Proc. Natl. Acad. Sci. U.S.A.* 103 6976–6981. 10.1073/pnas.0602171103 16636273 PMC1459004

[B37] Pang A. S. Nathoo S. Wong S. L. (1991). Cloning and characterization of a pair of novel genes that regulate production of extracellular enzymes in *Bacillus subtilis*. *J. Bacteriol.* 173 46–54. 10.1128/jb.173.1.46-54.1991 1898926 PMC207154

[B38] Parkhill J. Wren B. W. Mungall K. Ketley J. M. Churcher C. Basham D. (2000). The genome sequence of the food-borne pathogen *Campylobacter jejuni* reveals hypervariable sequences. *Nature* 403 665–668. 10.1038/35001088 10688204

[B39] Riddle M. S. Sanders J. W. Putnam S. D. Tribble D. R. (2006). Incidence, etiology, and impact of diarrhea among long-term travelers (US military and similar populations): a systematic review. *Am. J. Trop. Med. Hyg.* 74 891–900. 16687698

[B40] Ryan K. A. Karim N. Worku M. Penn C. W. O’Toole P. W. (2005). *Helicobacter pylori* flagellar hook-filament transition is controlled by a FliK functional homolog encoded by the gene HP0906. *J. Bacteriol.* 187 5742–5750. 10.1128/JB.187.16.5742-5750.2005 16077121 PMC1196087

[B41] Samuel M. C. Vugia D. J. Shallow S. Marcus R. Segler S. McGivern T. (2004). Epidemiology of sporadic *Campylobacter* infection in the United States and declining trend in incidence, FoodNet 1996-1999. *Clin. Infect. Dis.* 38(Suppl. 3), S165–S174. 10.1086/381583 15095186

[B42] Scharff R. L. (2012). Economic burden from health losses due to foodborne illness in the United States. *J. Food Prot.* 75 123–131. 10.4315/0362-028X.JFP-11-058 22221364

[B43] Silva J. Leite D. Fernandes M. Mena C. Gibbs P. A. Teixeira P. (2011). *Campylobacter* spp. as a foodborne pathogen: a review. *Front. Microbiol.* 2:200 10.3389/fmicb.2011.00200 PMC3180643 21991264

[B44] Sommerlad S. M. Hendrixson D. R. (2007). Analysis of the roles of FlgP and FlgQ in flagellar motility of *Campylobacter jejuni*. *J. Bacteriol.* 189 179–186. 10.1128/JB.01199-06 17041040 PMC1797208

[B45] Sundaram A. K. Pitts L. Muhammad K. Wu J. Betenbaugh M. Woodard R. W. (2004). Characterization of N-acetylneuraminic acid synthase isoenzyme 1 from *Campylobacter jejuni*. *Biochem. J.* 383 83–89. 10.1042/BJ20040218 15200387 PMC1134046

[B46] Tabei S. M. Hitchen P. G. Day-Williams M. J. Merino S. Vart R. Pang P. C. (2009). An *Aeromonas caviae* genomic island is required for both O-antigen lipopolysaccharide biosynthesis and flagellin glycosylation. *J. Bacteriol.* 191 2851–2863. 10.1128/JB.01406-08 19218387 PMC2668420

[B47] Toyama H. Fukumoto H. Saeki M. Matsushita K. Adachi O. Lidstrom M. E. (2002). PqqC/D, which converts a biosynthetic intermediate to pyrroloquinoline quinone. *Biochem. Biophys. Res. Commun.* 299 268–272. 10.1016/S0006-291X(02)02603-7 12437981

[B48] Toyama H. Nishibayashi E. Saeki M. Adachi O. Matsushita K. (2007). Factors required for the catalytic reaction of PqqC/D which produces pyrroloquinoline quinone. *Biochem. Biophys. Res. Commun.* 354 290–295. 10.1016/j.bbrc.2007.01.001 17223081

[B49] Vliet A. H. Ketley J. M. Park S. F. Penn C. W. (2002). The role of iron in *Campylobacter* gene regulation, metabolism and oxidative stress defense. *FEMS Microbiol. Rev.* 26 173–186. 10.1111/j.1574-6976.2002.tb00609.x 12069882

[B50] Wang Q. Suzuki A. Mariconda S. Porwollik S. Harshey R. M. (2005). Sensing wetness: a new role for the bacterial flagellum. *EMBO J.* 24 2034–2042. 10.1038/sj.emboj.7600668 15889148 PMC1142604

[B51] Weingarten R. A. Taveirne M. E. Olson J. W. (2009). The dual-functioning fumarate reductase is the sole succinate:quinone reductase in *Campylobacter jejuni* and is required for full host colonization. *J. Bacteriol.* 191 5293–5300. 10.1128/JB.00166-09 19525346 PMC2725595

[B52] Wösten M. M. Van Dijk L. Veenendaal A. K. De Zoete M. R. Bleumink-Pluijm N. Van Putten J. P. (2010). Temperature-dependent FlgM/FliA complex formation regulates *Campylobacter jejuni* flagella length. *Mol. Microbiol.* 75 1577–1591. 10.1111/j.1365-2958.2010.07079.x 20199595

[B53] Xia Q. Muraoka W. T. Shen Z. Sahin O. Wang H. Wu Z. (2013). Adaptive mechanisms of *Campylobacter jejuni* to erythromycin treatment. *BMC Microbiol.* 13:133 10.1186/1471-2180-13-133 PMC3694039 23767761

[B54] Young K. T. Davis L. M. Dirita V. J. (2007). *Campylobacter jejuni*: molecular biology and pathogenesis. *Nat. Rev. Microbiol.* 5 665–679. 10.1038/nrmicro1718 17703225

